# Improving treatment intensification to reduce cardiovascular disease risk: a cluster randomized trial

**DOI:** 10.1186/1472-6963-12-183

**Published:** 2012-07-02

**Authors:** Joe V Selby, Julie A Schmittdiel, Bruce Fireman, Marc Jaffe, Laura J Ransom, Wendy Dyer, Connie S Uratsu, Mary E Reed, Eve A Kerr, John Hsu

**Affiliations:** 1 Patient-Centered Outcomes Research Institute, Washington, DC, USA; 2 Division of Research, The Permanente Medical Group, Oakland, CA, USA; 3 Department of Medicine and Endocrinology, The Permanente Medical Group, South San Francisco, CA, USA; 4 Quality and Operations Support (formerly), The Permanente Medical Group, Oakland, CA, USA; 5 Center for Clinical Management Research, VA Ann Arbor Healthcare System, Ann Arbor, MI, USA; 6 Division of General Medicine, University of Michigan Medical School, Ann Arbor, MI, USA; 7 Mongan Institute for Health Policy, Massachusetts General Hospital, Boston, MA, USA; 8 Department of Health Care Policy, Harvard Medical School, Boston, MA, USA; 9 Kaiser Permanente Division of Research, 2000 Broadway, Oakland, CA, 94612, USA

**Keywords:** Diabetes mellitus, Hypertension, Hyperlipidemia, Cardiovascular diseases, Clinical inertia

## Abstract

**Background:**

Blood pressure, lipid, and glycemic control are essential for reducing cardiovascular disease (CVD) risk. Many health care systems have successfully shifted aspects of chronic disease management, including population-based outreach programs designed to address CVD risk factor control, to non-physicians. The purpose of this study is to evaluate provision of new information to non-physician outreach teams on need for treatment intensification in patients with increased CVD risk.

**Methods:**

Cluster randomized trial (July 1-December 31, 2008) in Kaiser Permanente Northern California registry of members with diabetes mellitus, prior CVD diagnoses and/or chronic kidney disease who were high-priority for treatment intensification: blood pressure ≥ 140 mmHg systolic, LDL-cholesterol ≥ 130 mg/dl, or hemoglobin A1c ≥ 9%; adherent to current medications; no recent treatment intensification). Randomization units were medical center-based outreach teams (4 intervention; 4 control). For intervention teams, priority flags for intensification were added monthly to the registry database with recommended next pharmacotherapeutic steps for each eligible patient. Control teams used the same database without this information. Outcomes included 3-month rates of treatment intensification and risk factor levels during follow-up.

**Results:**

Baseline risk factor control rates were high (82-90%). In eligible patients, the intervention was associated with significantly greater 3-month intensification rates for blood pressure (34.1 vs. 30.6%) and LDL-cholesterol (28.0 vs 22.7%), but not A1c. No effects on risk factors were observed at 3 months or 12 months follow-up. Intervention teams initiated outreach for only 45-47% of high-priority patients, but also for 27-30% of lower-priority patients. Teams reported difficulties adapting prior outreach strategies to incorporate the new information.

**Conclusions:**

Information enhancement did not improve risk factor control compared to existing outreach strategies at control centers. Familiarity with prior, relatively successful strategies likely reduced uptake of the innovation and its potential for success at intervention centers.

**Trial registration:**

ClinicalTrials.gov Identifier NCT00517686

## Background

Blood pressure, lipid, and glycemic control are essential for reducing cardiovascular disease (CVD) risk. [1-7] Although control of these conditions is improving nationally, [8-12] large proportions of patients remain suboptimally controlled. Non-adherence to prescribed medications is one reason for poor control. [13-15] “Clinical inertia,” or failure to advance pharmacotherapy in persons adhering to current medications, is another. [13,16-24]

Many health care systems have shifted aspects of chronic disease management to non-physicians with positive results for risk factor control. [25-28] Using treatment algorithms and electronic health information, pharmacists, nurses, or medical assistants can support patient self-management and advance evidence-based pharmacotherapy in collaboration with physicians.

In this translational study, we evaluated an enhancement of medical information used by non-physician outreach staff to further improve treatment intensification in patients at risk for CVD, who were adherent to current medications but with elevated blood pressure, LDL-cholesterol, and/or hemoglobin A1c values.

## Methods

This six-month intervention (July 1 - December 31, 2008) used a cluster randomized controlled design among enrollees of Kaiser Permanente Northern California (KPNC). The unit of randomization was KPNC medical center-based, non-physician, population management outreach teams (n = 8). KPNC is an integrated health care system providing comprehensive care to over 3.2 million enrollees through 21 medical centers. KPNC’s Institutional Review Board approved this study, waiving the requirement for individual informed consent because the intervention represented an enhancement to usual population-based outreach care.

### Study population

The KPNC PHASE (Preventing Heart Attacks and Strokes Everyday) population registry was identified in 2005 for a management program aimed at preventing CVD. [29] The registry is refreshed monthly and includes approximately 300,000 KPNC members, ages 18–85, at increased CVD risk because of: diabetes mellitus (DM); prior diagnoses of coronary artery disease, cerebrovascular disease, peripheral artery disease or abdominal aortic aneurysm; and/or advanced chronic kidney disease (age ≥ 50 and GFR < 30 or GFR <60 with proteinuria).

Medical center-based PHASE outreach teams are composed of a varying mix of clinical pharmacists, registered nurses, and medical assistants across centers. Teams conduct mail and telephone outreach to improve use of recommended medications and risk factor control. PHASE guidelines provide general evidence-based therapeutic algithms for achieving targets for blood pressure <130/80 mmHg for patients with DM or CKD <75 years, <140/90 mmHg for all others; LDL-cholesterol (LDL-c) <100 mg/dL; and hemoglobin A1c (A1c) <7.0%. For patients with prior CVD, aspirin (≥81 mg daily), a statin (≥40 mg simvastatin or equivalent daily), and an ACE-inhibitor (≥lisinopril 10 mg daily or equivalent) are recommended. For those with only DM, a statin from age 40, an ACE-inhibitor from age 55, and aspirin from age 50 in men and age 60 in women are recommended.

All teams use the Population Management Tool (PMT), a web-enabled database, to identify and track PHASE patients. The PMT presents risk factor and medication information that is refreshed weekly and accessible through standard queries. Some centers focus exclusively on reaching quality targets; others outreach to all patients periodically. Teams communicate with primary care physicians via the electronic health record, secure e-mail and telephone.

Center-level performance for risk factor control and use of recommended medications is monitored quarterly. Performance targets are one component of modest center-directed incentive payments[30].

### Identifying eligibility for treatment intensification

Within the PHASE population, risk factor levels and recent prescription fills were monitored monthly during the intervention to identify persons newly eligible for treatment intensification. The first search used a 3-month look-back window. Eligibility required: 1) most recent risk factor levels (systolic blood pressure (SBP), A1c, or LDL-c) above target (2 consecutive elevated values were required for SBP; 2) ≥ 7 months prior continuous KPNC enrollment to measure adherence and prior treatment intensification; 3) adherence to previously prescribed medications for the condition; and 4) no treatment intensification for the condition in the prior month. Blood pressures from inpatient, emergency, or procedure-related visits were not used to identify eligibility or assess effectiveness.

Adherence was measured separately for each condition by averaging adherence estimates across all condition-related medications in the period from 6 months before the elevated risk factor was noted to the date of the subsequent monthly data pull. For each medication, total days between the first prescription in this period and the data pull were summed and proportions of those days with available medications calculated. An average medication possession ratio was calculated across all condition-related medications. To account for stockpiling, remaining days supply from prior prescriptions for the same medication were added to numerators. Hospital days were subtracted from both numerators and denominators. Persons with average medication ratios of ≥ 80% were considered adherent and therefore eligible for treatment intensification. Patients on insulin but no oral diabetes medications at baseline were not considered for the A1c outcome because insulin adherence and treatment intensification are difficult to measure.

### Randomization, training and intervention process

The study concept was presented to PHASE team leaders in late 2007, along with data on the relationship of treatment intensification to improved risk factor control. [12] Leaders from nine out of a total of 17 center service areas expressed interest in participating. During the first 6 months of 2008, researchers met regularly with regional and center-based PHASE leadership to refine the study information to be placed in the PMT. In June, medical centers were randomized after blocking on center population size and current rates of treatment intensification. The small number of centers precluded additional blocking. Five centers were randomized to the intervention, four to the control arm. Before the intervention began, one intervention team underwent major re-organization and withdrew from the study. This left a total of 8 centers available for randomization.

Because of concern that staff might be unable to outreach to all eligible patients in a timely way, the study investigators and PHASE operations team leaders participating in the study created a prioritization scheme based on risk factor levels: Priority 1: SBP ≥ 140 mmHg; Priority 2: LDL-c ≥ 130 mg/dL; Priority 3: A1c ≥ 9%; Priority 4: SBP 130–139 mmHg in persons with diabetes or CKD; Priority 5: LDL-c 100–129 mg/dL; Priority 6: A1c 7–8.9%. For Priority 1, members ≤ 85 years were flagged. For other priorities, the upper age limit was 75. Staff at intervention centers agreed to process those in priorities 1–3 first. During the intervention, priority flags (up to 3 per patient), along with PHASE-recommended next steps in pharmacotherapy for each individual patient for use at the point-of-care, were prominently placed into the PMT and refreshed monthly. PHASE teams participating in the study were trained in the use of the new flags, and PHASE center leaders were provided with periodic feedback on how many eligible patients were being processed.

### Qualitatitive interviews

Qualitative pre- and post-intervention interviews with intervention team leaders at each center assessed goals, structure, and tactics of each center’s program, and recorded experiences and barriers encountered in using the new information. These interviews were conducted in the 2 months prior to the scheduled start of the intervention, and again after the intervention had ended. The baseline interview included domains to assess existing population care-based approaches to adherence and treatment intensification; staffing levels; and use of the PMT in existing PHASE program structure. The follow-up interviews included domains to assess changes in the population care programs as a result of the intervention; experience with the intervention; and barriers and facilitators to the intervention’s implementation at each site.

In addition, researchers met monthly with intervention team leaders during the study to support use of the new information. The PMT was modified to allow intervention staff to record dates and types of processing efforts (e.g. review, outreach).

### Analyses

Analyses were conducted separately by priority. Primary analyses considered Priorities 1–3. We excluded PHASE patients who did not have a KPNC pharmacy benefit and those in long-term care. Outcomes were receipt of treatment intensification within 3 months after placing an eligibility flag in the PMT (baseline), and changes in risk factor levels during 12 months post-baseline. Treatment intensification was defined as filling a prescription for a medication class not used in the prior 7 months or an increase in prescribed dosage of a current medication. Secondary analyses examined rates of intensification within 6 months and compared rates between intervention center patients processed by the outreach team and those not processed.

We used first recorded risk factor values at least 3 months post-baseline to assess mean risk factor change; we included all values for the entire 12-month post-baseline period in a repeated measures analyses. Although early post-baseline values in these analyses could precede treatment intensification, we included them because early values showing a return to control would be a reason not to order another test for some time. Small percentages of patients with no follow-up values of SBP, LDL-c, or A1c (3%; 14%, 10% respectively) were not included in analyses. Although we did not expect that treatment intensification or risk factor control would improve for priorities 4–6, we analysed these priorities in secondary analyses. We also compared proportions of patients “in control” during follow-up using priority-specific cutpoints in repeated measures analyses. Persons with missing values post-baseline were alternately treated as “not in control” or excluded. Finally, we compared control for the entire PHASE populations using last recorded values at 12 months after the intervention began.

Hierarchical logistic regression models (SAS PROC MIXED with GLIMMIX Macro) were used to compare treatment intensification rates, adjusting for patient age, sex, race/ethnicity, risk factor level and number of medications at baseline. Hierarchical linear regression models (SAS PROC MIXED) with an autoregressive covariance structure were used for repeated measures analyses of risk factor endpoints. A covariate for time from baseline to each measurement, and random effects for center, were included in all models.

In pre-study simulations (2500 iterations), we estimated study power to detect follow-up risk factor differences assuming 8 centers, 4 in each arm; 2300 eligible patients per center for blood pressure and LDL-c; 1700 for A1c control; average of 6 post-intervention blood pressure measures, one LDL-c and one A1c measure during 12-months follow-up. Intra-class correlations for centers were 0.01 for SBP, 0.006 for LDL-c, and 0.008 for A1c [31] . Simulations indicated 80% power to detect intervention-control differences of 3.4 mmHg for SBP; 6.7 mg/dL for LDL-c; 0.42% for A1c. The subsequent decision to focus only on patients in the top 3 priorities reduced eventual sample sizes by more than 50% for each risk factor, but focused on patients with most room for improvement.

## Results

**Figure 1:**
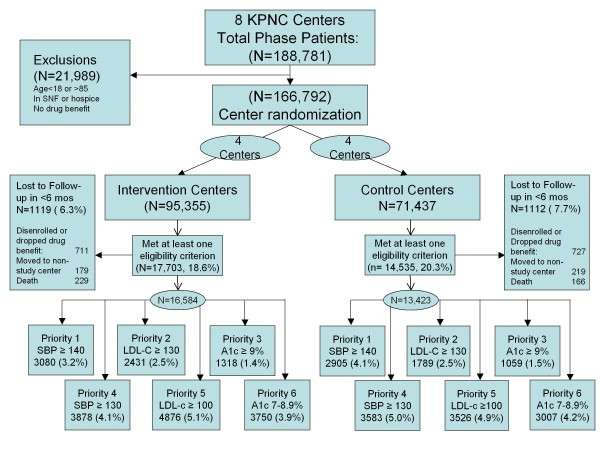
Flow sheet for cluster randomized trial showing total numbers of PHASE patients at participating medical centers, exclusions by reason, and final numbers of patients eligible at some time during the six-month intervention in each of six risk factor-based priority levels.

Of 188,781 total PHASE patients at participating centers, 11.6% were excluded because of age (n = 13,705), lack of a pharmacy benefit (n = 5,215), or residence in long-term care on July 1, 2008 (n = 3,069) (Figure 1). Of remaining patients, 18.6% of intervention center patients and 20.3% of control center patients met intensification criteria for at least one risk factor, and were also adherent to their medications for blood pressure (64% adherent), cholesterol (69% adherent) or diabetes (67% adherent) at some point during the 6-month intervention. Approximately 7% of subjects were subsequently excluded because of disenrollment or loss of a drug benefit; moving to a non-study medical center for primary care; or death in the first six months of follow-up. Among remaining eligible patients, fewer than half fell into the higher priorities 1–3 in each arm.

Intervention center patients were slightly older, less likely to be female, and much more likely to be white (62.0 vs. 44.4%) than control center patients (Table 1). Patients from control centers used slightly more blood pressure medications at baseline. Prevalence of PHASE-qualifying co-morbidities and smoking were similar. For the entire PHASE populations at these centers, control rates at baseline were high, especially for the levels of Priorities 1–3. Control was slightly better at intervention than control sites for SBP <140 mmHg but differed little for other priorities.

**Table 1:** Characteristics of Eligible Patient Population, Study vs. Control Sites

		**STUDY (n = 16,584)**	**CONTROL (n = 13,423)**	**P-Value**
Mean Age (yrs)		61	60	<.0001
% Female		49.9	51.8	<.0001
Race/Ethnicity (%)				
	Asian/PI	9.0	12.3	<.0001
	Black	6.1	16.2	<.0001
	Hispanic	9.8	12.6	<.0001
	Native American	1.3	1.3	0.97
	White	62.0	44.4	<.0001
	Other/Multiple	2.9	2.2	<.0001
	Missing Race	8.9	11.0	<.0001
Comorbidities				
	Mean Number of Comorbidities*	1.23	1.25	0.01
	Has Diabetes (%)	77.5	80.4	<.0001
	Current Smoker (%)	8.9	9.0	0.77
Mean Risk Factor Values at Baseline**				
	Systolic Blood Pressure (mm Hg)	131	132	0.18
	LDL-cholesterol (mg/dL)	100	98	<0.001
	Hemoglobin A1c (%)	7.3	7.3	0.13
Mean # Medication Classes, Baseline				
	Blood Pressure Medications	1.74	1.82	<.0001
	Diabetes Medications	1.22	1.23	0.19
	Dyslipidemia Medications	0.70	0.70	0.35
On Max Med Therapy at Baseline (%)				
	Simvastatin/Atorvastatin 80 mg	12.3	13.8	<.0001
	3 or more Blood Pressure Meds	27.8	31.0	<.0001
	Insulin, if has diabetes	20.0	20.5	0.38
Baseline control rates (%) Entire PHASE Populations - by priority level				
Priority 1: SBP <140 mmHg		83.5	81.8	
Priority 4: SBP <130 mmHg		61.1	60.7	
Priority 2: LDL-c <130 mg/dL		90.2	90.2	
Priority 5: LDL-c <100 mg/dL		71.8	71.7	
Priority 3: A1c <9%		90.3	90.9	
Priority 6: A1c <7%		60.5	60.0	

*Comorbidity count based on whether patient is flagged in the Population Management Tool as being in these populations: abdominal aortic aneurism, coronary artery disease, chronic kidney disease, diabetes, peripheral artery disease, and cerebrovascular disease.

### Treatment intensification

Treatment intensification within 3 months of eligibility was modestly but significantly more likely at intervention centers for Priority 1 (34.1 vs. 30.6%) and Priority 2 (28.0 vs. 22.7%) (Table 2). By six months, these differences had decreased and were not statistically significant (Priority 1: 47.2 vs.0 44.9%; Priority 2: 37.0 vs. 34.1%). Differences in treatment intensification for Priority 3 and for Priorities 4–6 were smaller and non-significant. Adjustment for baseline demographics, blood pressure or LDL-c values, and numbers of medications did not reduce the magnitude of either difference (33.4% vs. 29.4% for Priority 1; 31.5% vs 25.4% for Priority 2).

**Table 2:** Treatment Intensification Rates, Baseline Risk Factor Levels, Declines and Adjusted Differences During Follow-up - Study vs. Control Centers, Eligible Patients, by Priority Level

	**Study (n = 4 sites)**				**Control (n = 4 sites)**				
**Priority**	**Number Eligible Patients**	**Intensified within 3 months**	**Baseline Mean Risk Factor Level**^**†**^	**Unadjusted** **Risk Factor** **Decline**^**‡**^	**Number Eligible Patients**	**Intensified within 3 months**	**Baseline Mean Risk Factor Level**^**†**^	**Unadjusted** **Risk Factor** **Decline**^**‡**^	**Adjusted** **Study-Control Risk Factor Differences** **(95% CI)**^**¶**^
1: SBP ≥ 140	3,080	34.1^€^	153.2	13.6	2,905	30.6^€^	153.0	13.4	0.73
									(-1.40, 2.83)
2: LDL-c ≥ 130	2,431	28.0^€^	153.1	24.9	1,789	22.7^€^	153.9	23.8	-2.63
									(-6.73, 1.48)
3: A1c ≥ 9%	1,318	29.5	10.2	1.0	1,059	28.8	10.2	1.2	-0.02
									(-0.36, 0.32)
4:SBP 130-139^£^	3,878	22.9	138.5	6.0	3,583	22.0	138.5	5.0	-0.00
									(-1.25, 1.26)
5: LDL-c 100-129	4,876	20.5	111.4	10.1	3,526	19.1	111.3	8.9	-0.83
									(-4.10, 2.44)
6. A1c 7-8.9%	3,750	26.3	7.5	0	3,007	26.9	7.5	0	0.03
									(-0.10, 0.16)

^†^ Based on the last value recorded before follow-up begins; i.e., the value that qualified patient for treatment intensification.

^**‡**^ Based on the first value recorded after 3 months follow-up; if no values recorded between 3 and 12 months follow-up, the latest value recorded during first 3 months follow-up was used. No values were available during follow-up for 3%, 10%, and 14% of blood pressure, A1c, and LDL-c tests, respectively.

^**¶**^ From repeated measures models using all available values during follow-up,with adjustment for age, gender, race-ethnicity, baseline risk factor value, number of medications for the risk factor at baseline, and days of follow-up at time of each measurement. Negative value indicates that the adjusted difference favored the study group group during follow-up.

^€^ p < 0.001 for study vs. control comparison.

^£^ Only patients with diabetes mellitus and/or chronic kidney disease were eligible for the target of SBP <130 mmHg.

### Risk factor levels

Baseline risk factor levels were similar and post-baseline declines substantial at intervention and control sites, especially for Priority 1–3. No intervention-control site differences in adjusted risk factor levels were observed during follow-up (Table 2). The largest effect was seen for Priority 2, where intervention group subjects had adjusted LDL-c levels 2.6 mg/dL lower than control group subjects. In adjustment models for Priorities 2 and 3, addition of an indicator for treatment intensification within 3 months yielded large, statistically significant associations of treatment intensification with lower follow-up LDL-c and A1c levels. In contrast, receiving treatment intensification for SBP (Priority 1) was associated with significantly higher SBP values at follow-up, suggesting that, for blood pressure, intensification may have been reserved for those whose high SBP persisted through the early weeks of follow-up (rather than regressing to an earlier mean). After adjustment for baseline rates of treatment intensification, there was no evidence that the intervention led to greater heterogeneity in intensification rates than that seen among control centers.

Secondary analyses of proportions reaching control showed similarly small, non-significant differences for Priorities 1 and 2, whether patients with no follow-up values were treated as “not in control” or excluded. Over 50% or patients in each arm reached control. For priority 3, patients at control sites were more likely to be in control at follow-up (52.2% vs. 46.6%). This differences was somewhat smaller and no longer significant in the repeated measures analysis (4.9 mmHg, p = 0.22).

### Impact of intervention processing

Outreach staff at intervention sites initiated processing within 3 months for only 45 to 47% of patients in Priorities 1–3 (Table 3). Despite the intervention plan to process higher priority patients first, processing was also begun for 27 to 29% of patients in priorities 4–6. In each priority, treatment intensification was more frequent in patients for whom processing was begun. Differences were greatest for Priorities 2 and 5 (LDL-c control).

**Table 3:** Treatment Intensification Rates, Baseline Risk Factor Levels, Declines and Adjusted Mean Differences During Follow-up - Processed vs. Not Processed Patients, Intervention Centers Only, by Priority Level

		**Processed**			**Not Processed**			
**Priority**	**Percent Processed**^†^	**Intensified within 3 mos (%)**	**Baseline Mean Risk Factor Level**^**‡**^	**Unadjusted Decline**^**¶**^	**Intensified within 3 mos (%)**	**Baseline Mean Risk Factor Level**^**‡**^	**Unadjusted Decline**^**¶**^	**Adjusted Difference (Processed – not Processed) During Follow-up (95% CI)**^**€**^
1: SBP ≥ 140 mmHg	45	40.5	153.4	13.1	28.8	153.0	14.1	1.01
								(0.30, 1.75)
2: LDL-c ≥ 130 mg/dL	46	36.3	152.8	25.9	21.0	153.4	24.0	-0.6
								(-3.4, 2.2)
3: A1c ≥ 9%	47	32.6	10.3	1.0	26.7	10.2	1.0	-0.03
								(-0.19, 0.14)
4:SBP 130-139^£^	29	27.4	138.9	5.6	21.1	138.4	6.2	1.06
								(0.44, 1.69)
5: LDL-c 100-129 mg/dL	30	29.2	111.2	10.4	16.8	111.4	10.0	0.63
								(-0.95, 2.22)
6. A1c 7-8.9%	28	28.9	7.6	-0.1	25.3	7.5	-0.1	-0.03
								(-0.09, 0.04)

^†^ Processed: Record review and outreach initiated by staff in response to identification of eligibility for treatment intensification.

^**‡**^ Based on the last value recorded before follow-up begins; i.e., the value that qualified patient for treatment intensification.

^**¶**^ Based on the first value recorded after 3 months follow-up; if no values recorded between 3 and 12 months follow-up, the latest value recorded during first 3 months follow-up was used.

^**€**^ From repeated measures models using all available values during follow-up,with adjustment for age, gender, race-ethnicity, baseline risk factor value, number of medications for the risk factor at baseline, and days of follow-up at time of each measurement. Negative value indicates that the adjusted values were lower in the processed group during follow-up.

^£^ Only patients with diabetes mellitus and/or chronic kidney disease were eligible for the target of SBP <130 mmHg.

Baseline risk factor levels were similar between those who were processed and remaining patients. Processing was not associated with significant differences in risk factor declines during follow-up. For LDL-c, declines were slightly greater in those processed, but for SBP and A1C the converse was true.

### Control in entire phase populations

Control improved modestly in both intervention and control centers in the entire PHASE population for all priorities except Priority 6, where proportions with A1C <7% declined slightly for each group (Table 4). Changes were nearly identical for intervention and control populations for all priorities.

**Table 4:** Pre- and Post-Intervention Risk Factor Control Rates for Entire Phase Populations, by Study Arm and Priority Level

	**Study Facilities (n = 4)**			**Control Facilities (n = 4)**		
	PHASE Population Size ^1^	Pre-Intervention % in Control ^2^	Post-Intervention, % in Control ^2^	PHASE Population Size ^1^	Pre-Intervention % in Control ^2^	Post- Intervention % in Control ^2^
SBP ≥ 140	72,895	83.5	84.0	54,652	81.7	82.4
LDL-c ≥ 130	67,099	90.2	91.5	49,355	90.1	91.4
A1c ≥ 9%	48,464	90.9	91.0	38,497	90.3	90.7
SBP 130-139^¶^	72,895	61.1	62.8	54,652	60.7	62.6
LDL-c 100-129	67,099	71.8	75.3	49,355	71.7	75.5
A1c 7-8.9%	48,464	60.5	58.8	38,497	60.0	58.1

^1^ Members identified in Kaiser Permanente Northern California’s PHASE population from 1 year prior to the intervention to 1 year following the intervention, eligible for the priority by virtue of age and comorbidities, and with at least one risk factor measure in each period.

^2^ Control assessed based on latest recorded factor value during each period.

### Qualitative interviews with intervention site coordinators

All 4 intervention sites reported some continued pursuit of previous strategies that addressed risk factor control targets in all patients, without respect to study prioritization or adherence information. Staff reported feeling a need to continue focusing on their center’s quality targets, which covered the entire PHASE population of patients not currently in control. Two sites felt that the monthly PMT update was not timely enough and that staff were sometimes frustrated to find that treatment intensification had already been initiated by primary care physicians. The other two sites interpreted the same experience differently, suggesting that flags be placed in the PMT only after eligible patients had not received treatment intensification for three months, thereby focusing efforts on the smaller group in whom primary care appeared to have failed.

Some sites indicated that focusing on adherent patients, rather than all with poor control, was confusing. However, all 4 sites expressed interest both continuing to receive the treatment intensification information and also in receiving quantitative adherence information, believing both to be useful in working with patients not in control.

## Discussion

Meaningful use of electronic health information is a subject of ongoing study. We sought to reduce clinical inertia and enhance the efficiency of outreach efforts in the context of a successful population management program by focusing attention more closely on patients most likely to benefit from treatment intensification. The intervention added no new resources to clinical or outreach teams.

This translational intervention had at most a modest impact on likelihood of prompt treatment intensification, and for only two of three priority groups (hypertension and hyperlipidemia). It did not lead to improvements in risk factor levels compared to strategies already in place. Several prior studies of computerized reminders directed to physicians have yielded similar findings of improved care processes with little or no impact on risk factor levels [32-37], although one recent study did find positive results [38]. This may be the first report of use of such reminders for non-physician team members.

Within KPNC, risk factor control has improved steadily [12,30,31] likely due in substantial part to innovations in informatics and population management. Better-than- expected control rates at baseline and the decision to restrict the target population to patients with higher risk factor levels reduced sample size dramatically compared to that anticipated. More than 80% of all PHASE patients had blood pressures below the cutpoint for Priority 1 and over 90% were in control for Priorities 2 and 3 at baseline. Moreover, the small remaining proportions with poor control may be resistant to usual population management efforts, may have refused treatment intensification previously or been judged inappropriate for further intensification.

Despite reducing the target population, intervention outreach staff processed only 45-47% of high-priority patients, while also contacting 27-30% of lower-priority patients. Coupled with qualitative reports of difficulty integrating the new information into ongoing outreach and the need to achieve risk factor control for the population as a whole to achieve performance targets, these findings suggest that the context of an established, successful outreach program may have hindered full adoption. However, limited evidence suggested that if all patients had been processed, improvement in risk factor levels may have been seen, at least for lipid-lowering therapy.

Study limitations include the small numbers of medical centers randomized, making it impossible to block randomization on more than 2 variables. Consequently, some variables, including race/ethnicity, were not balanced. In addition, we were not able to measure socioeconomic status or use this variable to blance randomization or adjust the analysis. However, analyses adjusted for race/ethnicity, as well as for differences in baseline risk factor levels and numbers of medications used.

The study intervention had more limited reach than anticipated: teams were only able to reach less than half of the high-priority patients, and we were unable to collect detailed information on the processes teams used to contact patients (e.g. number of calls per patient). In addition, results from the qualitative interviews suggested that the information provided in the intervention may have been more useful if delivered more often than monthly. If the intervention had been more successful in reaching all targeted patients, and/or provided more timely updates, it is possible that we would have observed more favorable results.

Electronic health record and automated pharmacy data were used to determine adherence and intensification in this study, and due to the large volume of patients included in the intervention we were not able to validate this information via chart review, or to therefore include insulin adherence for diabetes patients. This potentially may have led to biases; however, many studies in this setting have relied on adherence and intensification information based on chart review and validated their relationship with CVD intermediate outcomes [12,13].

This study occurred in an integrated delivery system with high baseline control rates for CVD risk factors and well-developed non-physician population management outreach strategies. Findings may not generalize to other settings with lower baseline control rates or those employing different types of quality improvement strategies, where a similar intervention may have been more (or less) successful.

## Conclusion

In summary, enhanced information on need for treatment intensification did not improve risk factor levels in this population. High baseline control rates and a decision to narrow the target population limited the number of patients who could benefit from the intervention. Inadequate implementation further limited detectability of any potential benefits. Nonetheless, it is important to note that at study conclusion, outreach staff asked to continue receiving the new information, augmented by quantitative estimates of recent medication adherence for all patients. Future research should continue to focus on furthering our understanding of how to best integrated health IT tools into population management strategies to enhance the quality of care for patients with chronic conditions.

## Competing interests

The authors have no competing interests to declare.

## Author contributions

JVS, JAS, BF, and MJ conceived and designed the study. JVS, JAS, MJ, LJR, JH, MER, and EAK implemented the study at participating sites and contributed to the analysis of data. MER conducted the qualitative interviews. WD and CSU performed the statistical analysis. All authors have read and approved the final manuscript.

## Pre-publication history

The pre-publication history for this paper can be accessed here:

http://www.biomedcentral.com/1472-6963/12/183/prepub
